# Chemical Recycling of Bio-Based Thermosetting Epoxy Composite Produced by Vacuum-Assisted Resin Infusion Process

**DOI:** 10.3390/polym17091241

**Published:** 2025-05-02

**Authors:** Liberata Guadagno, Raffaele Longo, Marialuigia Raimondo, Luigi Vertuccio, Francesca Aliberti, Lorenzo Bonadies, Simone Morciano, Luigia Longo, Roberto Pantani, Elisa Calabrese

**Affiliations:** 1 Department of Industrial Engineering, University of Salerno, Via Giovanni Paolo II 132, 84084 Fisciano, Italy; rlongo@unisa.it (R.L.); mraimondo@unisa.it (M.R.); faliberti@unisa.it (F.A.); lbonadies@unisa.it (L.B.); rpantani@unisa.it (R.P.); elicalabrese@unisa.it (E.C.); 2 Department of Engineering, University of Campania “Luigi Vanvitelli”, Via Roma 29, 81031 Aversa, Italy; luigi.vertuccio@unicampania.it; 3 Research Center for Materials CFRC Technologies and Design (CETMA), S.S.7 Km.706 Via Cittadella della Ricercar, 72100 Brindisi, Italy; simone.morciano@cetma.it (S.M.); luigia.longo@cetma.it (L.L.)

**Keywords:** chemical recycling, epoxy resin, CFRCs

## Abstract

This research work focuses on the chemical recycling of a Carbon Fiber-Reinforced Composite (CFRC) manufactured through a vacuum-assisted resin infusion (VARI) process, characterized by a high Young’s modulus of approximately 7640 MPa. The recycling reaction was performed using a mixture of eco-sustainable solvents, composed of acetic acid and hydrogen peroxide, and was conducted at three different temperatures (70, 80, and 90 °C). The reaction yield values, evaluated with an innovative approach that involved the use of thermogravimetric analysis (TGA), confirmed the importance to recycle at a temperature corresponding to the glass transition temperature (Tg = 90.3 °C) of the resin. Spectroscopic investigations highlighted that the chemical bond cleavage occurred through the selective breaking of the C-N bonds of the cross-linked matrix structure, allowing the recovery of both the reinforcing phase of the epoxy matrix and the initial oligomers/monomers of the epoxy matrix. The morphological and electrical investigations carried out on the recovered fibers further confirmed the efficiency of the recycling process conducted at the highest explored temperature, allowing the recovery of cleaner fibers with an electrical conductivity value (8.04 × 10^2^ S/m) closer to that of virgin fibers (2.20 × 10^3^ S/m). The proposed strategy is a true challenge in terms of saving energy, solving waste disposal problems, preserving the earth, and preventing the depletion of planet resources.

## 1. Introduction

Over the last few decades, the widespread use of carbon or glass fiber-reinforced composites (CFRCs, GFRCs) in several fields, including the aeronautics [1,2], automotive, wind energy [3,4], and civil infrastructure fields [5], has led to growing concerns about the recycling of these materials to mitigate their environmental impact [6]. Recent research indicates that CFRC employment can be expanded further, thanks to the possibility of incorporating smart functions within the composite while preserving its lightness [7,8].

Fiber-Reinforced Composites (FRCs) consist of two main components: carbon or glass fibers, which serve as the ultralight, high-strength component, and a thermosetting polymeric matrix, typically made of epoxy resin. The current challenge for researchers is to recycle thermosetting composites by recovering both the carbon or glass fibers that serve as a reinforcement and the insoluble, infusible three-dimensional network of the thermosetting polymer, thereby converting this latest phase into monomers or oligomers [6,9].

The possibility of recovering the cured resin as a liquid mixture of monomers and oligomers opens up engaging scenarios that could lead to closed-loop recycling of the material, which is highly attractive due to its low environmental impact and reduced resource consumption.

Different recycling methods have been explored, including mechanical, thermal, and chemical recycling methods [10,11,12], to determine the most environmentally friendly and financially viable solution for waste management. All the recycling methods investigated have advantages and disadvantages; however, among them, the one that appears to be the most promising and that has yielded the best results is chemical recycling, which enables the recovery of both carbon fibers and the valuable building blocks of cured epoxy resin in the same process [6,9]. Typically, the chemical recycling of CFRCs involves the use of high temperatures and solvents with strong oxidizing power (e.g., nitric acid or hydrogen peroxide), conditions necessary to break the strong covalent chemical bonds that characterize the epoxy resin network impregnating the carbon fibers of the composite [13,14,15,16]. Alternative solutions involve the use of supercritical fluids, such as supercritical water [17,18,19,20,21,22] and supercritical alcohols [23], including methanol [24], ethanol, n-propanol [25,26], and i-propanol [27,28]. However, these approaches are detrimental to the environment as they produce toxic gases and require high energy consumption, with increased process costs. Furthermore, from a chemical perspective, they do not guarantee the selective breaking of chemical bonds, which would enable the partial preservation of the epoxy precursor’s structure, allowing for a greater possibility of reuse.

Very recent studies have focused on investigating the chemical recyclability of epoxy matrices as a sustainable disposable strategy, and they have also considered relevant aspects such as environmental benefits and economic advantages determined by the absence of any waste and the reduced need for virgin raw materials [29,30]. In particular, Saitta et al. investigate the chemical recycling of fully recyclable bio-based epoxy matrices based on epoxidized precursors derived from waste flour. The key to the epoxy matrix’s recyclability relies on using a cleavable hardener agent, which is cleavable in mild acetic conditions [29]. The study aims to identify re-use strategies in accordance with a circular economy approach. Cafaro et al. propose an optimized and sustainable chemical recycling method for epoxy resin matrices, which uses microwave-assisted reactions to completely recover the matrix without generating waste byproducts [30]. Together with the work performed on recycling epoxy matrices intended to produce composite materials, recent research has been performed on recyclable composites [31,32]. These research papers well reflect the pressing real need for “designs” of eco-friendly upcycling methods capable of effectively recovering and reusing the valuable carbon fibers and the polymer matrix from carbon-fiber reinforced polymer waste. Specifically, in this context, the attention is focused on cleavable components of CFRCs or on the possibility of transforming a decomposed polymer matrix into recyclable vitrimers [31,32]. Rossitti et al. used new commercial cleavable bio-resin formulations as a successful strategy to manufacture fully recyclable polymer composites [31].

Hao et al. propose an interesting approach for upcycling CFRP waste under environmentally friendly conditions. CFRP waste composed of an amine-cured epoxy resin was depolymerized in aqueous buffer solutions at a temperature ≤ 220 °C and an acid pH of 4.8. Further, the decomposed polymeric matrix was transformed from a conventional epoxy resin into recyclable vitrimers [32].

This research uses an efficient and cost-effective approach to recycle the CFRC produced by a vacuum-assisted resin infusion (VARI) process. The recovery reaction occurs through selective C-N bond cleavage, enabling the recovery of carbon fibers and the epoxy material’s backbone in a single step. The chemical recycling process was conducted under very mild conditions, using an environmentally friendly solvent mixture of acetic acid and hydrogen peroxide at atmospheric pressure and temperatures below 100 °C. Three reaction temperatures (70, 80, and 90 °C) were examined to evaluate the relationship between the resin’s glass transition temperature (Tg = 90 °C) and depolymerization temperature. FT-IR spectroscopy was employed to demonstrate the selective cleavage of the C-N bond, and the thermogravimetric analysis technique enabled the accurate evaluation of the depolymerization yield. Morphological and electrical analyses of the recycled fibers confirmed the effectiveness of the recycling reaction performed at a maximum temperature of 90 °C, highlighting the significance of operating at the Tg of the resin to reduce the rigidity of the polymer chains and enhance the swelling and oxidation actions of the solvent mixture. The work performed combines three relevant specific conditions: (a) the epoxy matrix of the composite is composed of a bio-based epoxy precursor and a bio-based amine hardener, the resin is 36% bio-based, and the hardener is free of phenol and phenol-related chemicals; (b) no catalytic products were used; (c) low temperatures were selected for the depolymerization.

Hence, the conditions of the depolymerization reactions, which are conducted at low temperatures (lower than 100 °C) and without catalysts, allow for reducing energy consumption and recovering the resin in liquid form without further steps to eliminate products due to degradation and catalysis. Thus, the recovered resin is ready for reutilization.

Furthermore, a very innovative approach, adopting thermogravimetric analysis, was proposed to rapidly evaluate the yield of the depolymerization reaction.

## 2. Materials and Methods

### 2.1. Materials

#### Carbon Fiber-Reinforced Composite

The epoxy mixture used for the infusion was composed of a bio-based epoxy precursor (trade name: Resin M1049) and a biobased amine hardener (trade name: hardener M2049) provided by WS Marine s.r.l. (Genova, Italy), an Italian distributor of Pro-Set products (Burlington, WA 98233, USA), an Italian distributor of Pro-Set products (Genova, Italy). The resin is 36% bio-based, and the hardener is free of phenol and phenol-related chemicals. It is studied to provide fast and thorough wetting of the reinforcing carbon fabric within the laminate during infusion. Data concerning the bio-based epoxy components are shown in Section S1 of Supplementary Materials (S.M.); see Tables S1–S5.

A biaxial carbon fiber MATES BXC 400 45 (HS) was used. It was provided by MATES Italiana (Milano, Italy). The main physical parameters are shown in Section S1 of Supplementary Materials (S.M.) (see Table S6).

**Figure 1:**
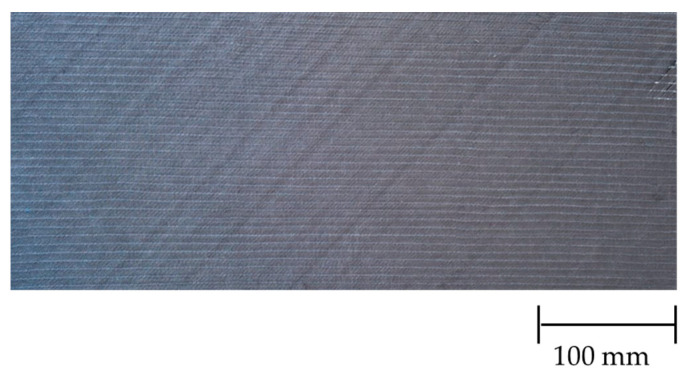
Optical image of the CFRC.

The composite was produced using a vacuum-assisted resin infusion (VARI) process. Infusion CFR techniques typically rely on the pressure differential principle between the resin source and the vacuum chamber (cavity) [33]. The VARI process involved impregnating a woven carbon fabric with resin, where the liquid resin was drawn into a dry laminate and maintained under a vacuum against a rigid glass surface, enclosed by a sealed, flexible film. The detailed procedure for manufacturing the composite is described in Section S2 of Supplementary Materials (S.M.) (see Figures S1 and S2). The optical image of the manufactured CFRC is shown in Figure 1.

### 2.2. Methods

#### 2.2.1. Mechanical Characterization

Mechanical characterization was performed on the formulated composite using a Dual Column Tabletop Testing System (Series 5967, with a 30 kN load cell) provided by Instron (Norwood, MA 02060-2643, USA) in both tensile and bending modes. The mechanical tests were performed on five samples with a rectangular geometry (1.4 × 15 × 50 mm^3^) at a 1.0 mm/min displacement rate.

Furthermore, dynamic mechanical analyses (DMAs) were conducted to evaluate the mechanical properties of the cured EP resin in the temperature range from 25 °C to 200 °C using a DMA 8000 provided by PerkinElmer (Waltham, MA 02451, USA). The test was performed in compression mode at a frequency of 1 Hz with an amplitude of 0.5 mm on a rectangular sample (5 × 10 × 1.2 mm^3^) at a scanning rate of 1 °C min^−1^.

#### 2.2.2. Fourier Transform Infrared (FT-IR)

The spectroscopic analyses were performed on the oligomers recovered through the recycling of the CFRC. The FT-IR spectra were obtained in absorbance using a Bruker Vertex 70 FT-IR spectrophotometer with a resolution of 2 cm^−1^ (32 scans collected) in the 4000–400 cm^−1^ range. The FTIR spectrum of the cured epoxy-based formulation was collected by dispersing the sample powder in KBr pellets, which served as carriers. The FT-IR spectra of the oligomers were collected by spreading viscous samples of the depolymerized resin on the KBr pellet. Section S3 of Supplementary Materials (S.M.) show the FT-IR signals assignment (see Table S7).

#### 2.2.3. Thermogravimetric Analyses (TGA)

TGA measurements were conducted using a TGA/DSC 3+ Star System thermal analyzer, with the experiments performed under a nitrogen flow. The weight loss as a function of temperature was recorded at a rate of 10 °C/min from 30 to 700 °C. All measurements have been performed using samples with a minimum weight of 9 mg.

#### 2.2.4. Scanning Electron Microscopy/Energy-Dispersive X-Ray Spectroscopy (SEM/EDS)

The recovered fibers obtained from the depolymerization reaction and the virgin fibers were analyzed by Scanning Electron Microscopy (SEM) as previously reported in other research papers for various types of fibers [34,35]. The SEM image was acquired using a Phenom ProX microscope operating in high-vacuum mode. The depolymerized fibers and virgin fibers were metalized with a layer of gold using an Agar Automatic Sputter Coater (Mod. B7341, Stansted, UK) at 40 mA for 180 s. EDS information was obtained by using an EDS tool from the same machine.

#### 2.2.5. Electrical Characterization

The electrical measurements were performed using 4-wire sensing to obtain accurate conductivity values, following a procedure described in a previous paper [36]. The generator used is EA Elektro Automatik EA-PSI 9500-10 T (Helmholtzstr, 41747 Viersen, Germany). The multimeter used for recording the voltage at the center of the sample is the HP 34401A Digital Multimeter (Palo Alto, CA, USA).

#### 2.2.6. X-Ray Diffraction (XRD)

The structural organization of the samples was analyzed using wide-angle X-ray diffraction (WAXD) patterns in reflection mode, which were collected by using a Bruker D2 Phase diffractometer (Bruker, Billerica, MA, USA) operating at 35 kV and 40 mA (CuKα radiation X-ray source, λ = 0.15418 nm) and equipped with Lynxeye (1D mode) detector. The increment of theta has been set at 0.01517°, while the time step was 0.100 s.

#### 2.2.7. Morphological Investigation by Tunneling Atomic Force Microscopy (TUNA)

The electrical conductivity of the virgin and recycled fibers obtained through depolymerization reactions was analyzed at the nanoscale level using the TUNA technique. TUNA is a contact mode technique in which the tip is in uninterrupted contact with the sample. The measurements require an electrically conductive tip of 20 nm and platinum-coated probes with nominal spring constants of 35 N·m^−1^. During the measurements, the DC sample bias was set to values between 1 and 3 V, which are really low if we consider that the limit for this parameter is 12 V; the current sensitivity was 1 pA/V; the scan rate was set to 1.00 Hz s^−1^, halving the value to 0.500 Hz s^−1^ to improve the image quality; the number of samples per ramp was 256, increasing to 512 to obtain higher-resolution images. It is worth noting that, due to the high values of the electrical conductivity of the manufactured panel, the characterization by TUNA was carried out without grounding the samples. Usually, for this type of measurement carried out on polymeric materials, it is necessary to use the conductive silver paste, which ensures adequate electrical contact of the sample with the ground. The images of Deflection Error and TUNA current, collected simultaneously, were examined using the Bruker software Nanoscope Analysis 1.80 (BuildR1.126200). The Deflection Error image represents the error signal of the deflection parameter that defines the required voltage (and, therefore, the required deflection or force of the cantilever) for the feedback circuit. The Deflection error is closely related to the deviation of the vertical deflection from the deflection setpoint generated when the tip comes into contact with a particle during the scanning phase of the sample surface.

In these conditions, the tip undergoes a slight rebound upwards, causing a slight upward bending of the cantilever with a consequent increase in vertical deflection. However, the feedback circuit can effectively act by returning the vertical deflection to its nominal value using the gain set by the user and sent to the piezo Z to move the tip up or down to minimize the error. TUNA Current images give a map of the distribution of conductive electrical domains at the atomic scale, thus demonstrating its extreme sensitivity in detecting currents as low as in the order of the femtoampere.

## 3. Results

### 3.1. Mechanical Characterization

#### 3.1.1. Stress–Strain Curves

**Figure 2:**
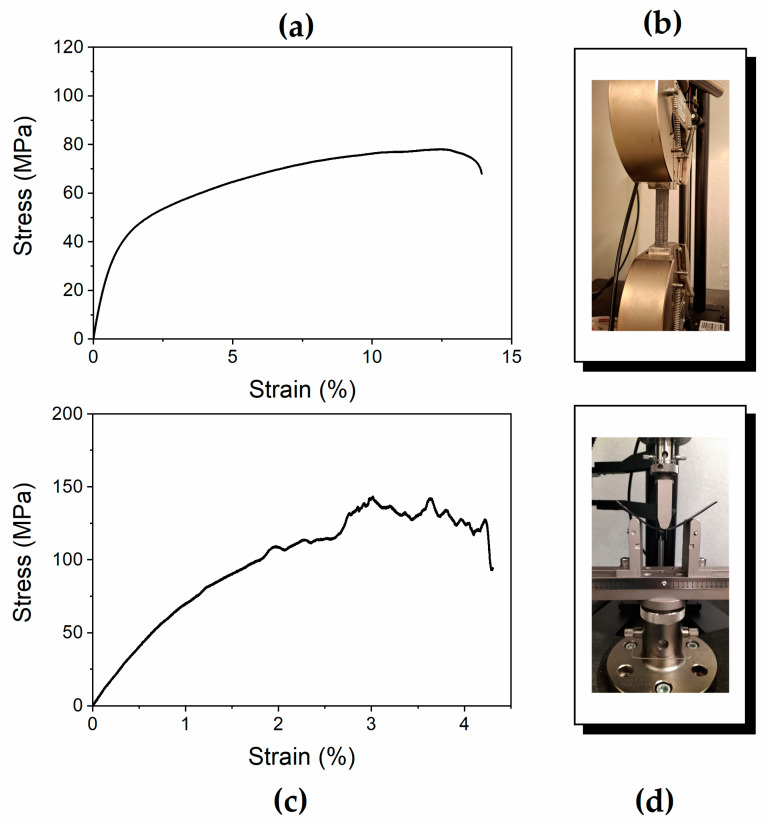
Results of stress–strain tests: (**a**,**b**) tensile tests, (**c**,**d**) bending tests.

Figure 2 shows the stress–strain curves of one of the five tested specimens obtained by performing measurements in both tensile and three-point bending modes (see Figure 2a,c, respectively). In particular, Figure 2b,d show the specimen positioned within the instrument, ready for the test. The Young’s modulus, stress, and elongation at break values of all the specimens are reported in Table 1 and Table 2, together with the average and standard deviation values.

The tensile test results indicate that Young’s modulus values are approximately 7 GPa, and the range of deformability of the material increases to 13% when the composite fractures (see Table 1). The mechanical modulus obtained from bending tests, which is generally used to characterize the flexural properties of structural laminates, corresponds to 9.3 GPa (see Table 2). These results demonstrate that the composite exhibits mechanical properties characteristic of materials suitable for structural applications.

#### 3.1.2. DMA Tests Performed on the Cured Epoxy Resin

**Figure 3:**
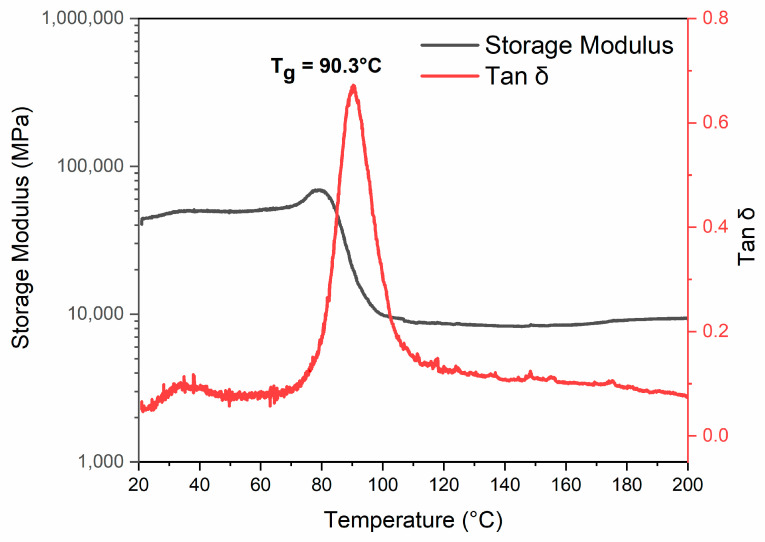
Results of DMA tests of EP sample: evolution of storage modulus (black curve) and loss factor (Tan δ, red curve) as a function of temperature.

DMA analysis was conducted on the cured epoxy resin EP to determine the material’s glass transition temperature (Tg). Knowing the temperature range at which the material starts the glass transition is fundamental information for choosing the temperature to perform the depolymerization reaction as, during the transition’s step, a reduction in the polymeric chains’ rigidity occurs, and the solvent’s swelling action is promoted, ensuring a more efficient oxidizing action of the solvents. The results of the DMA analysis, shown in Figure 3, indicate that the Tg of the cured resin, determined as the maximum of the Tanδ peak, is 90.3 °C, with a transition onset temperature of approximately 70 °C. These values led to investigating three possible recycling temperatures—70, 80, and 90 °C—to evaluate the depolymerization efficiency at values close to the Tg of the material and lightly lower than it.

### 3.2. Chemical Recycling of the Composite

**Figure 4:**
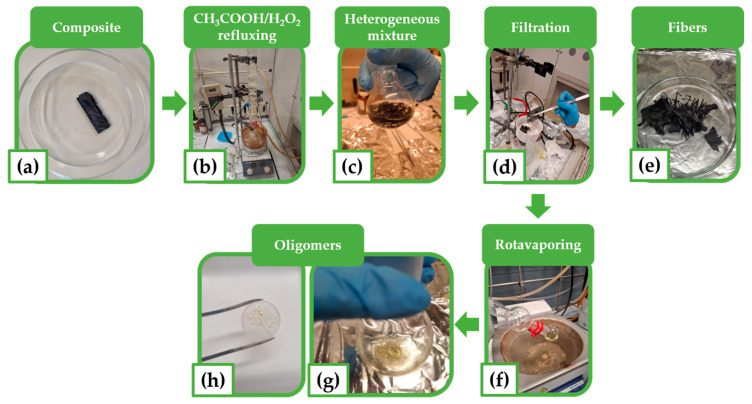
Optical images illustrating the phases of the experimental recycling procedure: (**a**) piece of composite subjected to depolymerization; (**b**) solution of CH_3_COOH/H_2_O_2_ for depolymerization in the flask equipped with a reflux condenser; (**c**) resulting heterogeneous mixture; (**d**) filtration of resulting heterogeneous mixture; (**e**) recovered fibers; (**f**) rotavaporating; (**g**) recovered viscous oligomers: (**h**) viscous oligomers spread on a KBr tablet and ready for FT-IR analysis.

The resin used for the composite infusion was an amine-cross-linked bio-based resin, and Figure 4 illustrates the steps of the recycling experimental procedure. A panel of the composite was cut into individual pieces to obtain the samples subjected to depolymerization. Three samples with a rectangular geometry (30 mm × 10 mm) were obtained. One of these is shown in Figure 4a. The samples were chemically treated at three different temperatures—90 °C, 80 °C, and 70 °C—labeled as CFRC-90, CFRC-80, and CFRC-70, respectively. The reaction conditions adopted for the recovery of the fibers were chosen based on studies present in the literature concerning similar research activities [9,37]. The thermoset composite was introduced into a 50 mL glass reaction flask, and the solvent mixture was added at a solvent-to-sample mass ratio of 60 mL/g.

The solvent mixture used for depolymerization was a solution of acetic acid (CH_3_COOH, 14 M) and hydrogen peroxide (H_2_O_2_, 9 M or 30% *w*/*w*), mixed in a weight percentage of 95:5. The flask was equipped with a reflux condenser. The reaction mixture was stirred and heated for 6 h (see Figure 4b). The resulting heterogeneous solution (see Figure 4c) was filtered through a Buckner funnel (see Figure 4d) to separate the liquid phase from the recovered fibers (see Figure 4e). The recovered fibers were washed with distilled water until a neutral pH was reached; they were washed with acetone and dried in a vacuum oven at 100 °C for 4 h. The solvent was removed under vacuum by a rotavapor from the liquid solution containing the depolymerization products (the oligomers) (see Figure 4f). The oligomers were recovered as a yellow, viscous liquid (see Figure 4g) and characterized by FT-IR spectroscopy (see Section 3.3). It is worth noting that the optical image of Figure 4h, showing the viscous oligomers spread on a KBr tablet and ready for FT-IR analysis, is aimed to attest to the physical aspect of the recovered oligomers.

The depolymerization yields obtained at the selected temperatures are illustrated in Table 3. Typically, the matrix dissolution efficiency is evaluated by calculating the resin decomposition ratio, based on an equation reported in the literature that includes, as a key parameter, the mass fraction of the resin in the initial composite. In this research, the authors propose a new alternative method based on thermogravimetric evaluations. Specifically, TGA curves were used to assess the percentage of resin present in the composite piece after the cutting and chemical recycling process. Then, the method considers the amount of the resin still attached to the recovered fibers. Following this new approach, the depolymerization Yield (Dy) was calculated according to Equation (1):

(1) $$D_{Y}=\left(\frac{Y_{0}-Y_{1}}{Y_{0}}\right)\times 100$$

where Y_0_ is the resin mass fraction on the initial composite and Y_1_ is the resin mass fraction on the recovered fibers. The values of Y_0_ and Y_1_ were determined by evaluating the weight loss percentages of the tested samples, as obtained through the TGA analyses described in Section 3.4.1. These values were determined by using the equations systems (2) and (3), reported below:

X_0_ + Y_0_ = 1 (at 30 °C) X_0_ + αY_0_ = Z_0_  (at 550 °C) (2)

X_1_ + Y_1_ = 1 (at 30 °C) X_1_ + αY_1_ = Z_1_  (at 550 °C) (3)

where X_0_ and X_1_ are the fiber mass fractions in the initial composite and the recovered fibers, respectively, while Z_0_ and Z_1_ are the values of the TGA residuals at 550 °C for the composite and the recovered fibers, respectively (see the TGA curves in Section 3.4.1). The constant α is the TGA residual at 550 °C for the cured resin EP, and its numeric value (0.119) is reported as a percentage in Table 4 of Section 3.4.1, together with the Z_0_ and Z_1_ values. By using the equations system (2) for the composite (CFRC), it was possible to determine the percentage of resin present in the pristine composite (Y_0_) equal to 56.1%, while, by applying the equations system (3) for the recovered fibers, it was possible to evaluate the amount of resin still anchored to the recovered fibers, corresponding to the Y_1_ values, illustrated (as percentages) in Table 3.

The depolymerization reaction, carried out at 90 °C, resulted in a yield of 81.3%, with nearly complete fiber recovery. In correspondence with the resin’s Tg, the recycling process yields the most promising results due to a reduction in the rigidity of the polymeric chains and more efficient swelling of the resin in the solvent. Furthermore, a more effective oxidant action is promoted in this condition due to the higher solvent-to-resin ratio inside the composite. The optical images of the recycled fibers r-F70, r-F80, and r-F90 are reported in Figure S3 of the Supplementary Materials (S.M.). They attest that only the fibers recovered at 90 °C appeared well separated, while those recovered at lower temperatures were still compact, as only the separation of the layers constituting the material occurred due to a higher amount of the resin that was still adherent to the fibers. The results of the morphological investigation, reported in Section 3.4.2, are in perfect agreement with these results.

### 3.3. Characterization of the Liquid Recovered Epoxy Mixture

#### FT-IR Analyses

**Figure 5:**
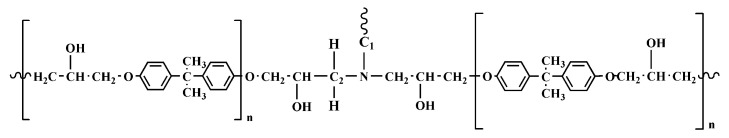
Chemical structure of the epoxy-based network.

FT-IR analyses were performed to characterize the oligomers obtained from the depolymerization procedure and to study the degradation mechanism. The chemical structure hypothesized, considering all the spectroscopic data, is schematically represented in Figure 5. It is very similar to the amine-cross-linked DGEBA resin. This hypothesis is confirmed by the relevant similarity between the FTIR spectra of the two epoxy-based formulations in a wide analyzed spectral range (see Figure S4 and Table S6) in Section S3 of the Supplementary Materials (S.M.).

As previously described, the chemical recycling of the CFRC resulted in the separation of the carbon fibers from the epoxy resin, which was broken down into oligomeric fragments, likely through selective cleavage of the C-N bond, leaving the structure of the epoxy precursor almost intact. Peracetic acid can oxidatively break down cross-linked epoxy resin in a one-step process, operating through a mechanism that cleaves C-N bonds. The epoxy-based network shows two aliphatic C-N bonds (see Figure 5); the first one connects the nitrogen atom to the hardener moiety (C_1_-N), and the second one binds the nitrogen atom to the epoxy precursor chain (C_2_-N): both or just one of them could break during the chemical treatment of the fiber-reinforced composite, determining the formation of oligomers having carbonyl functional groups. The signals of the FT-IR spectra of the recovered depolymerized resin matrix of the CFRC perfectly agree with this hypothesis. Concerning the depolymerization of the thermosetting matrix, it was demonstrated that FT-IR investigation is very effective in understanding the chemical composition of the depolymerized products, among which functional groups and the nature of the oligomers [38,39,40].

**Figure 6:**
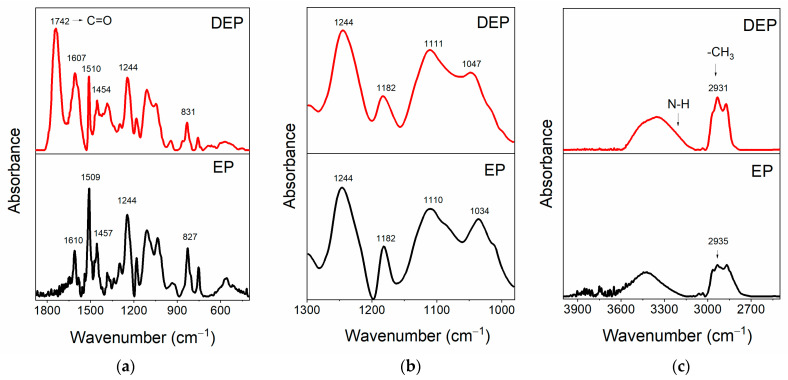
FT-IR spectra of the samples EP and DEP, in the range of wavenumbers: (**a**) 1880–400 cm^−1^; (**b**) 1300–980 cm^−1^; (**c**) 4000–2500 cm^−1^.

Figure 6 compares the FT-IR spectra of the cured epoxy resin EP alone (see black curve) and the oligomers obtained after the composite was chemically treated at 90 °C for 6 h, indicated by the acronym DEP (see red curve).

**Figure 7:**
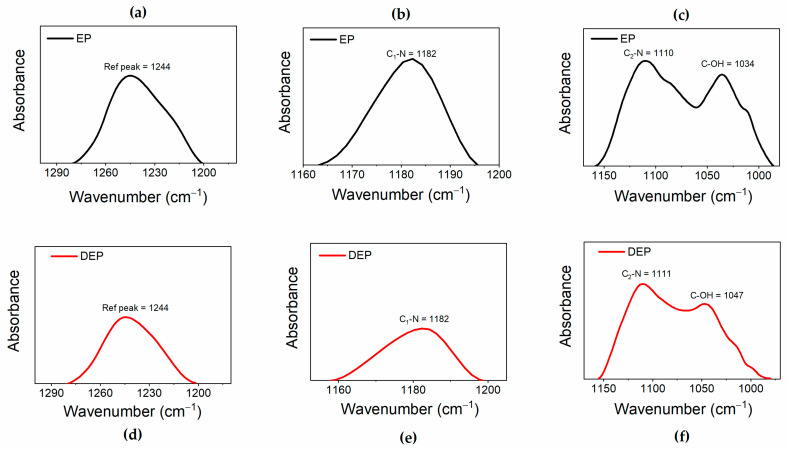
Normalized FT-IR bands for (**a**–**d**) reference peak; (**b**–**e**) C_1_-N peak; (**c**–**f**) C_2_-N; and C-OH peaks.

This comparison highlights that depolymerization allows the preservation of the main structure of the resin. Figure 6a shows that, in the FT-IR spectra of both the samples, it is possible to detect the peaks belonging to the benzene skeleton (stretching vibration of benzene ring) at 1610-1509-1457-827 cm^−1^ for the EP sample, and at 1607, 1510, 1454, and 831 cm^−1^ for the products of the depolymerization DEP. The signal at 1244 cm^−1^ in EP is ascribed to the stretching vibration of C-O-C, and this signal, attributed to aryl alkyl ethers (aryl-O-CH_2_-), also appears in the spectrum of recycled depolymerization products, indicating the conservation of aryl alkyl ether [41,42]. The presence of the band at 1742 cm^−1^ only in the DEP sample spectrum indicates the formation of an ester carbonyl group in the depolymerized products [39]. The enlargement in the region of wavenumbers between 1300 and 980 cm^−1^, illustrated in Figure 6b, allows for a better observation of the C-N and C-O bands. In particular, the peaks at 1182 cm^−1^ and around 1110 cm^−1^ can be attributed to C_1_-N bond and C_2_-N, respectively, while the signals at 1034 cm^−1^ for EP, shifted to 1047 cm^−1^ for DEP, belong to C−OH of diglycidyl ether unit [39,43]. To better analyze the variations affecting the C-N and C-O bonds, which could be involved in the depolymerization process, the changes in the intensities of the peaks associated with these bonds were evaluated with respect to a reference peak. The reference peak chosen is that of the aryl ethers, which is assumed to be unchanged during the depolymerization. To correctly evaluate peaks’ intensities, the latter were subjected to a baseline correction procedure, as shown in Figure 7, for the FT-IR bands of the reference peak (see Figure 7a–d), and of the C_1_-N (see Figure 7b–e), C_2_-N, and C-OH (see Figure 7c–f) bands.

**Figure 8:**
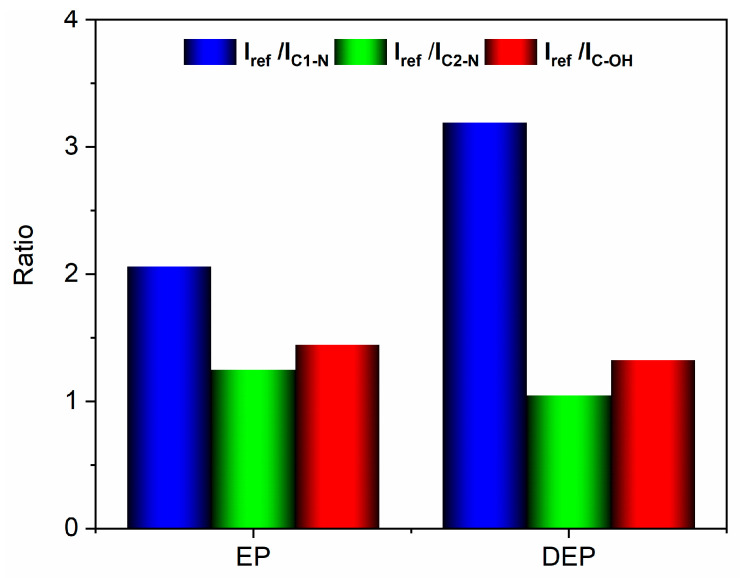
Histogram with the intensity ratio values.

The variations in the ratios between the intensities of the reference peak and the other peaks, observed in the analyzed samples, have been considered diagnostic for evaluating the bonds involved in the degradation reaction of the cured epoxy resin. The results are reported in the histogram of Figure 8, which illustrates the ratios between the intensity of the reference peak and the C_1_-N peak (I_ref_/I_C1-N_), the C_2_-N peak (I_ref_/I_C2-N_), and the C-OH peak (I_ref_/I_C-OH_), for both the analyzed samples.

**Figure 9:**
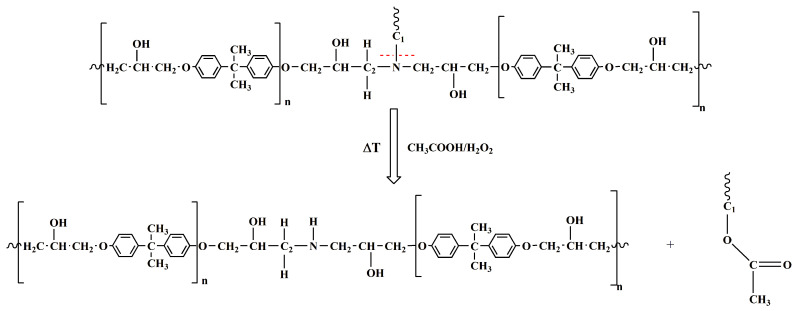
Scheme illustrating the supposed reaction mechanism for the degradation process.

The histogram indicates that the I_ref_/I_C1-N_ ratio increases, suggesting a decrease in the signal intensity associated with the C_1_-N bond in the depolymerized products. The other two ratios remain almost unchanged. These results mean that the depolymerization reaction most probably involves the cleavage of the C_1_-N bond that connects the epoxy precursor unit to the hardener moiety. This cleavage occurs due to an attack by the acyloxy radical (CH_3_COO.) that could lead to the regeneration of the N-H bond, as also suggested by the presence of a broad band in the wavenumber region between 3600 and 3100 cm^−1^, that may contain the N-H stretching vibration of a secondary amine (see red curve of Figure 6c) [39]. Furthermore, an intensification of the signal related to the stretching of −CH_3_ at 2931 cm^−1^ is observed in the DEP spectrum (2935 cm^−1^ for the EP sample, see Figure 6c), due to the presence of a greater amount of methyl groups, following the action of the acyloxy radicals generated from peracetic acid. The supposed depolymerization products are reported in Figure 9.

The results confirm a selective cleavage of the C-N bond, most likely corresponding to the C_1_-N bond, with the consequent formation of ester groups and secondary amino groups. This mechanistic hypothesis was also confirmed by the investigation through nuclear magnetic resonance (NMR) spectroscopy, the results of which are reported in the Section S4 of Supplementary Materials (S.M.) (see Figures S5–S7).

### 3.4. Characterization of the Recycled Fibers

#### 3.4.1. TGA Analyses

**Figure 10:**
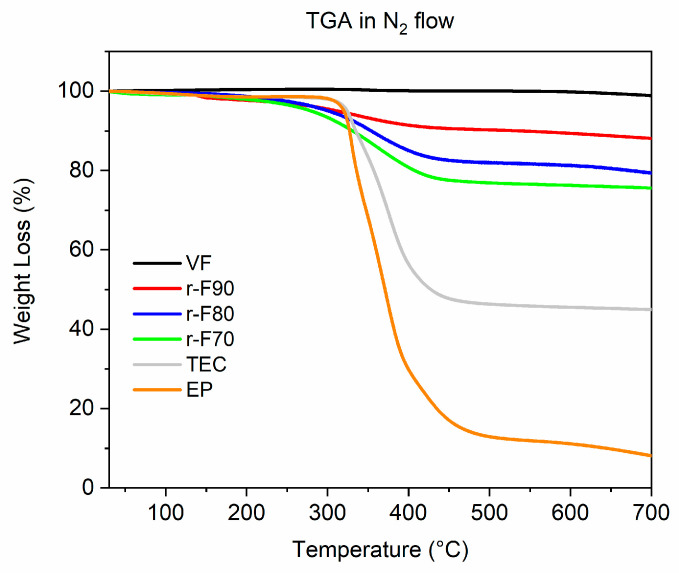
Thermogravimetric curves of the analyzed samples.

A TGA investigation was conducted to evaluate the thermal behavior of the tested samples and to determine the Y_1_ and Y_0_ parameters for calculating the D_Y_ value, as reported in Section 3.2. Figure 10 illustrates a comparison of the thermograms for the virgin fiber, cured resin, and recovered fibers.

Table 4 provides an overview of the TGA results, including the T_d5%_ and T_d50%_ values, which represent the temperatures at which 5 wt% and 50 wt% of the material’s mass is lost. 

**Table 4:** TGA data.

Sample	T_5%_ (°C)	T_50%_ (°C)	Residual at 550 °C (%)		
			α	Z_0_	Z_1_
EP	320.2	370.8	11.9	-	-
TEC	324.2	427.5	-	45.9	-
r-F70	279.6	743.2	-	-	76.5
r-F80	301.3	795.4	-	-	81.7
r-F90	311.9	758.9	-	-	89.9

T_d5%_, known as the onset degradation temperature, is typically used to assess the thermal stability of a material during degradation [44]. 

The TGA profile of the VF shows that the sample does not undergo any thermal degradation phenomenon up to 700 °C, with a residue equal to 100%. The thermogram of the pristine resin EP highlights the thermal degradation behavior is mainly characterized by a single decomposition stage, that realizes around 350 °C, and a similar trend can be observed in the TGA curves of the CFRC and of the recovered fibers, where the main degradation step can be attributed to the thermal decomposition of the resin present in the sample. Regarding the TGA curves of the recovered fibers, the higher the temperature at which depolymerization occurs, the lower the thermal decomposition drop in the TGA curves of the depolymerized samples. This behavior aligns perfectly with the observation that increasing the depolymerization temperature increases the depolymerization yield. Furthermore, the T_d5%_ values indicate a decrease in the initial degradation temperature for the recovered fibers compared to the untreated composite and the pristine resin. These results highlight that the resin remaining on the recovered fibers is a residual resin that, being partially decomposed by the chemical treatment, degrades at lower temperatures.

#### 3.4.2. SEM/EDS Analyses

**Figure 11:**
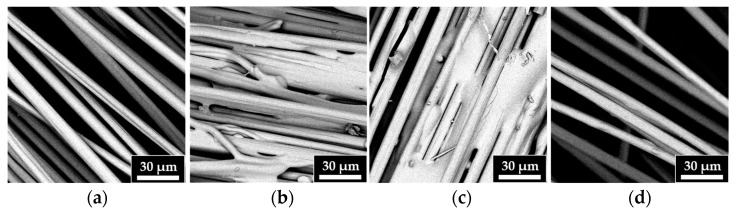
SEM images of (**a**) VF, (**b**) r-F70, (**c**) r-F80, and (**d**) r-F90.

SEM images, shown in Figure 11, allow for the observation of the morphology of the recycled fibers (Figure 11b–d) compared to that of the virgin fibers (Figure 11a). The results highlight that increasing the reaction temperature results in cleaner fibers, particularly in the surface layer of the resin. The fibers recovered at 90 °C show a morphology similar to that of the virgin fibers.

**Figure 12:**
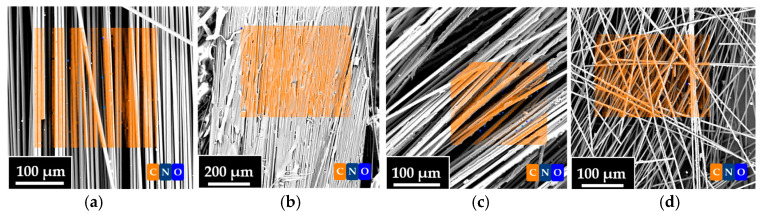
Combined SEM and EDS images of (**a**) VF, (**b**) r-F70, (**c**) r-F80, and (**d**) r-F90.

Furthermore, EDS analyses were carried out to evaluate the elemental composition of the fibers. Figure 12 and Table 5 show the combined results of SEM/EDS images. In particular, Figure 12 shows the SEM images of the VF, r-F70, r-F80, and r-F90 samples. The transparent orange box superimposed on the SEM images represents the surface area used for the EDS investigation for each sample. Table 5 shows the element percentage composition of the characterized samples. From Table 5, it is evident that the carbon content of the recycled fibers increases as the temperature of the depolymerization reaction increases. The weight concentration values of the carbon atom show that the carbon content for the r-F90 sample (72%) is very close to that of virgin fibers (75%). 

##### Statistical Evaluation of Fibers’ Diameters

**Figure 13:**
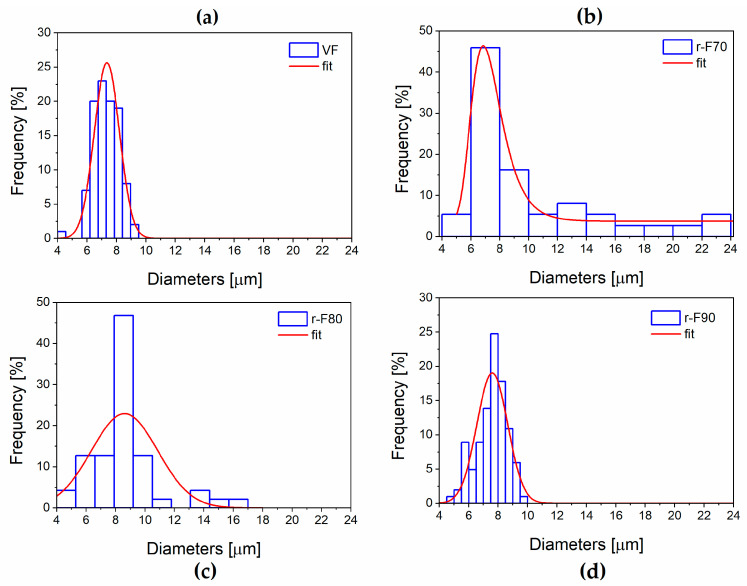
Fibers’ diameter distribution: (**a**) VF; (**b**) r-F70; (**c**) r-F80; (**d**) r-F90.

**Figure 14:**
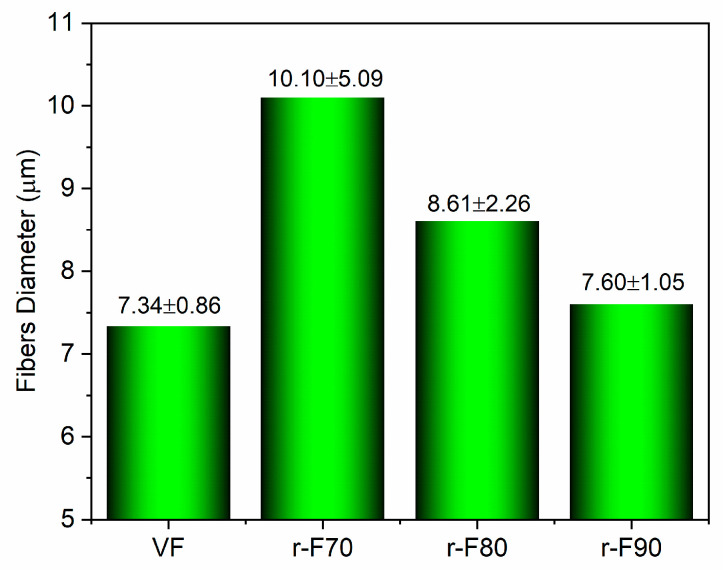
Histogram reporting fibers’ diameters.

It is worth noting that the resin attached to the fibers could also affect their diameters. For this reason, the average diameter of the recycled fibers was statistically evaluated using SEM image processing, performed with ImageJ^®^ software (Version 1.54) (see Figure 13 and Figure 14). Figure 14, which reports the histogram of fiber diameters, demonstrates that the higher the resin residual, the higher the fiber diameter, increasing from 7.34 μm (VF) to 10.10 μm (r-F70). The residual resin causes an increase in the fiber diameter distribution, also leading to a rise in the standard deviation of the fiber diameter dimensions. Furthermore, by evaluating the diameter of depolymerized fibers obtained at the temperature of 90 °C, it is observed that the average diameter and standard deviation are comparable with those of the virgin fiber.

#### 3.4.3. Electrical Characterization

**Figure 15:**
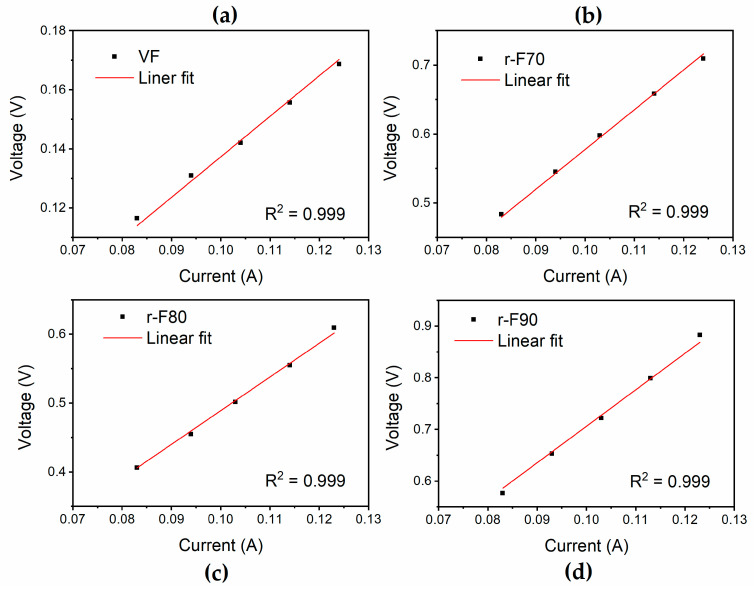
Results of the electrical voltage–current tests for (**a**) VF, (**b**) r-F70, (**c**) r-F80, and (**d**) r-F90.

To further demonstrate the efficiency of the recycling process, electrical conductivity measurements were performed on the recycled fibers and compared to those of the virgin fibers. The results of the analyses, as reported in Figure 15, indicate that the materials exhibit ohmic behavior. The electrical conductivity values, shown in Table 6, are higher for the fibers recovered at 90 °C, as this temperature removes a more significant amount of the insulating resin from the fibers’ surface, making them progressively less insulating. In particular, the r-F90 sample exhibits the highest electrical conductivity value among the recycled fibers, almost comparable to that of virgin fibers.

#### 3.4.4. TUNA Investigation

The TUNA method was used to study the electrical features of the virgin and recycled fibers. Thanks to the conductive nanodomains, the local tunneling current is recorded while the probe scans in contact mode. More specifically, the TUNA Current image scale bar shows a range of colors, from the darkest for less conductive areas to the lightest for more conductive regions, which indicate the degree of local electrical conductivity.

**Figure 16:**
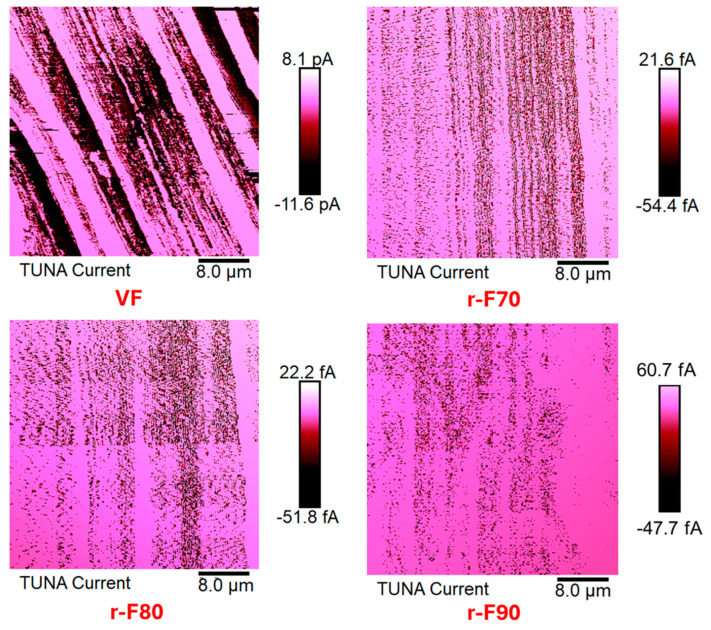
TUNA Current micrographs of virgin fibers (VF) and recycled fibers r-F70, r-F 80, and r-F90.

**Figure 17:**
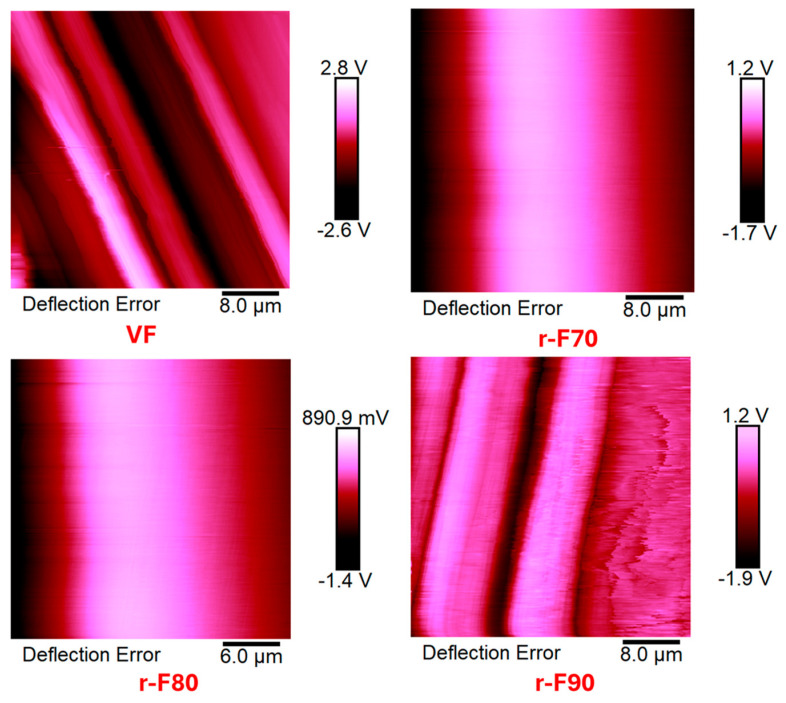
Deflection Error micrographs of virgin fibers (VF) and recycled fibers r-F70, r-F80, and r-F90.

The research activity results perfectly agree with the occurrence that the resin content around the CFs decreases as the depolymerization temperature increases. Figure 16 shows TUNA current micrographs of the virgin carbon fiber VF and the depolymerized samples (r-F70, r-F80, and r-F90), while Figure 17 shows Deflection Error micrographs that highlight greater morphological details for the same samples.

From TUNA Current images of Figure 16, it is observed that the r-F70 sample shows current values ranging from −54.4 to 21.6 fA, which are low compared to the current values between −11.6 and 8.1 pA observed for the virgin fibers VF. Notably, the electrical conductivity values at the nanoscale level exhibit the same trend as the electrical DC conductivity, which is approximately one order of magnitude lower (2.23 × 10^2^ S/m) compared to the VF sample (2.20 × 10^3^ S/m). For sample r-F90, which has the least resin attached to the fibers, the electrical conductivity increases to 8.40 × 10^2^ S/m. In fact, for this sample, from TUNA electrical characterization, current values between −47.7 and 60.7 fA were recorded, which are higher than those recorded for the fibers depolymerized at 70 °C and 80 °C.

In particular, the morphological images in Figure 17 clearly show how the recycled fibers at 70 and 80 °C are still entirely covered by resin, while the fibers treated at 90 °C emerge on the surface without clearly showing the thick coverage of the resin layer. This evidence is more marked in the Deflection Error images.

#### 3.4.5. XRD Analyses

**Figure 18:**
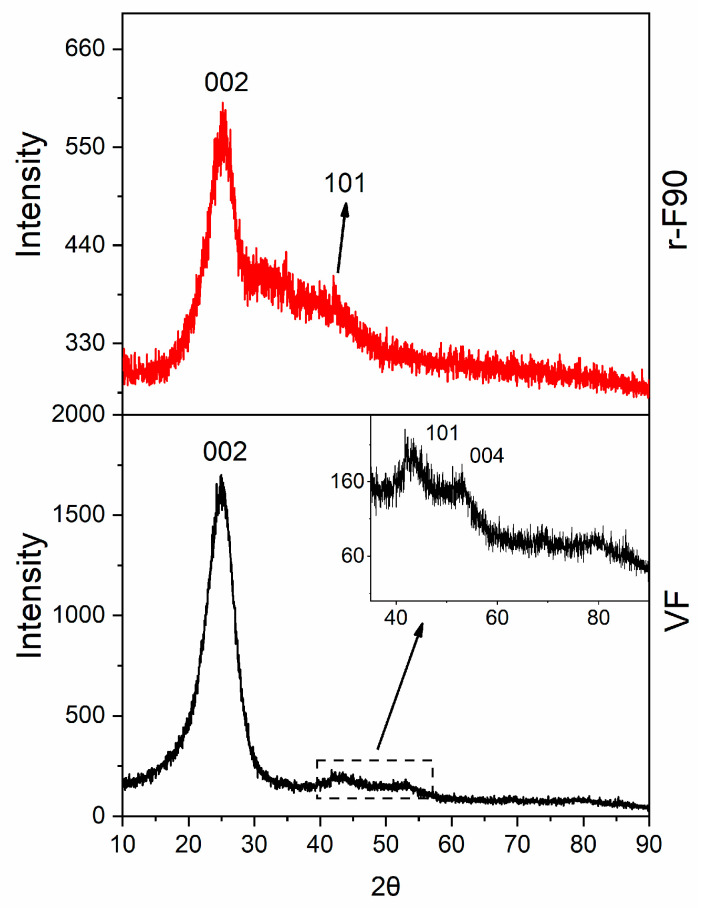
X-Ray pattern of: VF (black curve) and r-F90 (red curve).

The results collected from the three temperatures selected for the depolymerization clearly indicate that 90 °C is the optimal temperature for achieving a high depolymerization yield while maintaining a low depolymerization temperature (below 100 °C). The X-ray pattern of the sample depolymerized at 90 °C, r-F90, is depicted in Figure 18, alongside that of the virgin fiber VF, included for comparison.

The XRD profile of the virgin fiber shows an intense and narrow 002 graphite reflection, together with the weaker reflections 101 and 004. The observed reflections indicate the presence of hexagonal graphite with ABAB stacking of layers, which agrees with what is reported in the literature for similar carbon fibers [5]. The same reflections are also detectable in the XRD pattern of the recycled fibers r-F90, which preserve their crystallinity.

The lower intensity and slight broadening of the reflections of the recycled fibers are due to the presence of a low amount of residual resin still anchored on the fiber surface (the reflections are overlapped by the large amorphous halo of the resin fraction) and the different arrangement of the fibers (more unpacked than the virgin ones).

## 4. Conclusions

The experimental activity demonstrated that it is possible to recycle high-performance thermosetting composites under mild temperature and pressure conditions using low-impact solvents. The TGA-based method, employed for evaluating the degree of depolymerization, accurately allows for calculating the quantity of resin impregnating the fibers in the starting composite, and that which is still anchored to the fibers after the recycling procedure. The values of depolymerization yield increase with increasing temperature used during the chemical treatment of the composites. The highest yield value was obtained by treating the composite at a temperature of 90 °C for 6 h. This result demonstrates the importance of chemically treating the composite at a temperature corresponding to the resin’s Tg (equal to 90.3 °C), thereby reducing the rigidity of the polymeric chains and promoting the swelling of the resin due to the solvent action. FT-IR spectroscopic analyses revealed that using a mixture of eco-friendly solvents, such as acetic acid and hydrogen peroxide, enabled the selective cleavage of C-N bonds, leaving the skeleton of the oligomeric chains intact. Morphological analyses confirmed the success of the depolymerization reactions, indicating that the amount of resin anchored to the fibers decreases as the temperature of the chemical treatment increases. Finally, the electrical conductivity evaluation at the macroscopic and nano-metric levels of the recycled fibers further demonstrated the efficiency of the material’s recovery process. The results of this research work are promising, as they could lead to a friendly and efficient recycling process that is easily transferable to an industrial scale, both for existing composite materials from air or naval fleets and for new composites that will constitute the materials to be recycled in the future.

## Figures and Tables

**Table 1:** Tensile mechanical parameters.

Sample	Young Modulus (MPa)	Strain at Break (%)	Stress at Break (MPa)
Test 1	6635.4	12.2	80.1
Test 2	8986.2	16.4	93.6
Test 3	7491.9	13.5	77.0
Test 4	8043.9	17.5	87.7
Test 5	7041.4	13.9	78.0
Average	7639.8	14.7	83.3
Standard Deviation	917.2	7.1	2.2

**Table 2:** Bending mechanical parameters.

Sample	Young Modulus (MPa)	Strain at Break (%)	Stress at Break (MPa)
Test 1	8864.5	2.79	172.2
Test 2	9530.7	3.08	168.7
Test 3	9798.6	2.96	199.3
Test 4	9214.3	3.44	164.9
Test 5	8891.6	3.01	159.3
Average	9259.9	3.1	172.9
Standard Deviation	405.5	0.2	15.5

**Table 3:** Reaction conditions and yields.

Sample	Temperature (°C)	Y_1_ (%)	D_Y_ (%)
CFRC-70	70	26.6	56.6
CFRC-80	80	20.8	66.2
CFRC-90	90	11.5	81.3

**Table 5:** Element percentage composition of the characterized samples.

Sample	Carbon (C)	Nitrogen (N)	Oxygen (O)
VF	75.12	16.37	8.51
r-F70	63.96	24.81	11.23
r-F80	65.77	19.87	14.36
r-F90	71.83	15.07	13.09

**Table 6:** Electrical conductivity data.

Sample	σ (S/m)	Standard Deviation (S/m)
VF	2.20 × 10^3^	12.9
r-F70	2.23 × 10^2^	1.55
r-F80	3.19 × 10^2^	2.81
r-F90	8.04 × 10^2^	9.58

## Data Availability

The data presented in this study are available upon request from the corresponding author due to privacy reasons.

## Supplementary Materials

The following supporting information can be downloaded at https://www.mdpi.com/article/10.3390/polym17091241/s1, Figure S1: Black carbon preform overlapped by the peel ply and the net; Figure S2: Schematic representation of the process for the CFRP manufacturing; Figure S3: Optical images of the recovered fibers: (a) r-F70; (b) r-F80; (c) r-F90; Figure S4: Comparison between the FT-IR spectra of the EP sample and an amine-cured DGEBA sample in the wavenumber region of 1800–400 cm^−1^; Figure S5: ^1^H-NMR spectrum of the degradation product DEP; Figure S6: ^13^C-NMR spectrum of the degradation products DEP; Figure S7: DEPT 135 spectrum of the degradation products DEP; Table S1: Mix ratio; Table S2: Density; Table S3: Handling Properties; Table S4: Mechanical Properties; Table S5: Thermal Properties; Table S6: Physical parameters of Carbon Woven Fabric; Table S7: FT-IR signals assignment.
