# Supplementary material for: Chemical Recycling of Bio-Based Thermosetting Epoxy Composite Produced by Vacuum-Assisted Resin Infusion Process
Source: Polymers (Basel). 2025 May 2;17(9):1241. doi: 10.3390/polym17091241 (PMC12073523; doi:10.3390/polym17091241)
Supplement: Supplementary file 1 [file polymers-17-01241-s001.zip › polymers-3602436-supplementary.pdf]

## Supplementary Electronic Materials

# Chemical Recycling of a Bio-Based Thermosetting Epoxy Composite Produced by Vacuum-Assisted Resin Infusion Process

**Liberata Guadagno [1,*], Raffaele Longo [1], Marialuigia Raimondo [1], Luigi Vertuccio [2], Francesca Aliberti [1], Lorenzo Bonadies [1], Simone Morciano[3], Luigia Longo[3], Roberto Pantani[1], Elisa Calabrese [1]**

Department of Industrial Engineering, University of Salerno, Via Giovanni Paolo II 132, 84084 Fisciano, Italy; mraimondo@unisa.it (M.R.); rlongo@unisa.it (R.L.); elicalabrese@unisa.it (E.C.); faliberti@unisa.it (F.A.); lbonadies@unisa.it (L.B.)

[2] Department of Engineering, University of Campania "Luigi Vanvitelli", Via Roma 29, 81031 Aversa, Italy; luigi.vertuccio@unicampania.it (L.V.)

[3] Research Center for Materials Technologies and Design (CETMA), S.S.7 Km.706 Via Cittadella della ricerca, 72100 Brindisi, Italy

* Correspondence: lguadagno@unisa.it; Tel. +39 089 964114

### *Section 1*

### *Epoxy Blend Mixing Ratio and Related Properties*

Data on the Mixing Ratio (Resin/hardener), resin, and handling properties are provided in Tables S1-S5.

**Table S1.** Mix ratio.

| Method | Resin: Hardener | Resin: Hardener |
|---|---|---|
| weight | 2.94:1 | 100:34 |
| volume | 2.44:1 | 100:41 |

**Table S2.** Density.

| State | Units | Reference temperature: 21°C |
|---|---|---|
| Mixed | g $cm^{-3}$ | 1.11 |
| Resin | g $cm^{-3}$ | 1.17 |
| Hardener | g $cm^{-3}$ | 0.96 |

**Table S3.** Handling Properties.

| Property | Standard | Units | Reference Temperature 25°C |
|---|---|---|---|
| 100g Pot Life | ASTM D2471 | minutes | 136 |
| 500g Pot Life | ASTM D2471 | minutes | 90 |
| 3mm thin film gel time | | minutes | 1375 |
| Viscosity Mixed | ASTM D2196 | mPa s | 220 |
| Viscosity (resin) | ASTM D2196 | mPa s | 480 |
| Viscosity (hardener) | ASTM D2196 | mPa s | 25 |

**Table S4.** Mechanical Properties.

| Property | Standard | Units | RT x 24 hrs + 50°C x 16 hrs | RT x 24 h + 50°C x 16 h+ 80°C x 2 h |
|---|---|---|---|---|
| Hardness | ASTM D2240 | Shore D | 82 | 83 |
| Compression Yield | ASTM D695 | MPa | 86.8 | 85.4 |
| Tensile Strength | ASTM D638 | MPa | 58.4 | 45.8 |
| Tensile Modulus | ASTM D638 | GPa | 3 | 3 |
| Tensile Elongation | ASTM D638 | % | 4.5 | 8.8 |
| Flexural Strength | ASTM D790 | MPa | 96 | 88.4 |
| Flexural Modulus | ASTM D790 | GPa | 4.4 | 4.1 |

**Table S5.** Thermal Properties.

| **Property** | **Standard** | **Units** | **RT Gelation + 50°C x 16 hrs** | **RT Gelation + 50°C x 16 h + 80°C x 2 h** |
|---|---|---|---|---|
| Tg DMA Peak Tan Delta | ASTM E1640 (1Hz, 3°C per minute) | °C | 74.2 | 83 |
| Tg DSC Onset – 1st heat | ASTM E1356 | °C | 52.6 | 56.6 |
| Tg DSC Ultimate | ASTM E1356 | °C | 68.3 | 71.7 |

*Physical parameters of Carbon Woven Fabric*

The carbon fiber woven fabric is biaxial, +/- 45°, in 400 g/m$^2$ 50k HS carbon fiber.

Data on the Physical parameters of Carbon Woven Fabric are provided in Table S6.

**Table S6.** Physical parameters of Carbon Woven Fabric.

| **Test** | **Units** | **Value** | **Normative** |
|---|---|---|---|
| Mass per unit area | gr/sqm | 405 (±5.0%) | ISO 3374 |
| Weave | Type | Biaxial | ISO 2113 |
| Tensile Strenght | MPa | 4137 | ISO 14130 |
| Tensile Modulus | GPa | 242 | ASTM D 790 |

***Section 2***

***Composite Manufacturing: Detailed Procedure***

The composite was prepared using a Lay-Up procedure, which involves manually laying down individual plies of carbon fiber wovens. This process involves manipulating each ply into shape by hand and then firmly sticking them to the previous layer or mold surface, leaving no air pockets between plies. In this research activity for the composite manufacturing, three plies of carbon fabric (210 x 480 mm$^2$) were employed. They

were placed on a glass shelf to support the composite fabrication. The glass shelf was previously cleaned with acetone and a specific "mold cleaner". Afterwards, an adhesive paper tape was placed on the edges of the glass support, which was covered with a liquid release agent (TR45ECO). Only when the surface was completely dry was the 3-layer perform placed on the shelf.

Once the mold preparation was completed, the resin/hardener mixture was prepared, as reported in the technical data sheet, with a mass ratio of epoxy precursor to hardening agent equal to 100/34. For the mixing ratio, it is important to consider the "pot life" (workability time) of the resin. The "pot life" is the time for which a certain quantity of resin, at a specific temperature, remains workable before hardening, that is, before undergoing an increase in viscosity that negatively affects the manufacturing process. The technical data sheet indicates that the resin's " pot life " decreases as the mixture's mass quantity increases (e.g., pot life for 100 g = 136 min, pot life for 500 g = 90 min).

After placing the carbon preform on the glass support, two spirals were positioned on the shorter edges of the preform and fixed with butyl tape to the resin inlet and outlet side. Then, the carbon preform was overlapped with a white peel ply and a blue net, acting as a distribution medium (see Figure S1). The function of the peel ply is to facilitate the escape of air bubbles and vapors trapped in the preform towards the outlet tube. The net must cover the same points as the peel ply, ensuring the resin flows uniformly over the preformed overall. The synergistic effect between the peel ply and the net allows the elimination of voids during the process.

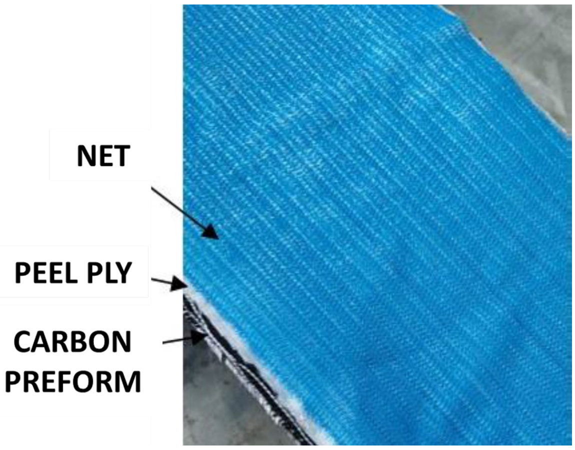


**Figure S1.** Black carbon preform overlapped by the peel ply and the net.

Afterward, two tubes were positioned at the shorter sides of the preform, and their ends were connected on one side to the resin trap, which was connected to the vacuum pump, and on the other side to the resin reservoir, as shown in the scheme reported in Figure S2.

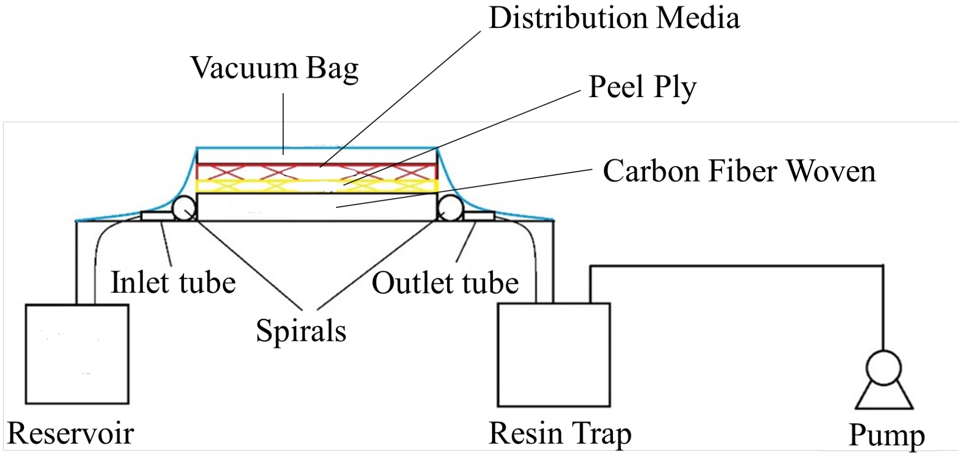

**Figure S2.** Schematic representation of the process for the CFRP manufacturing.

Finally, the vacuum bag was applied through a gluing procedure. Once the bag was completed, a vacuum seal test was performed, using a ΔP of -0.9 bar, which lasted 15 minutes. Once the bag seal was verified, the infusion phase was performed. The entire infusion process lasted about 13 minutes. Subsequently, the vacuum pressure was reduced to ΔP = -0.7 bar to aspirate any residual bubbles. The reagent mixture was left to cure at room temperature for 24 hours. Once the curing process at room temperature was completed (before extraction from the vacuum bag), the composite was subjected to a post-cure treatment consisting of two steps: a first step was performed at 50°C for 16 hours, and a second step at 80°C for 2 hours.

*Section 3*

*Optical images of the recovered fibers*

The optical images of the recovered fibers at temperatures of 70 °C r-F70 (a), 80°C (b), and 90°C are shown in Figure S3.

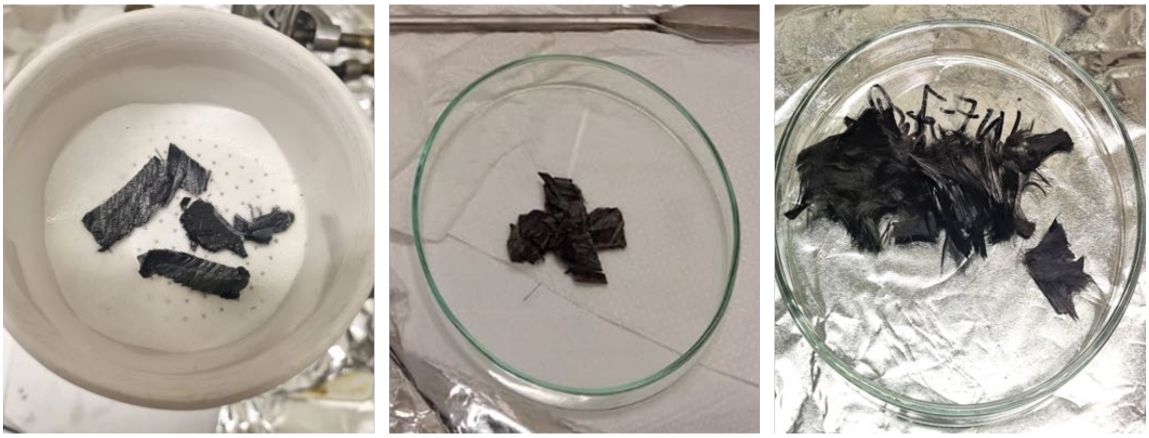

**(a)** **(b)** **(c)**

**Figure S3.** Optical images of the recovered fibers: (a) r-F70; (b) r-F80; (c) r-F90.

*Comparison between the FT-IR spectra of the EP sample and an amine-cured DGEBA sample*

Figure S4 presents a comparison of the FT-IR spectra of the EP sample with an amine-cured DGEBA sample in the wavenumber range of 1800-400 $cm^{-1}$.

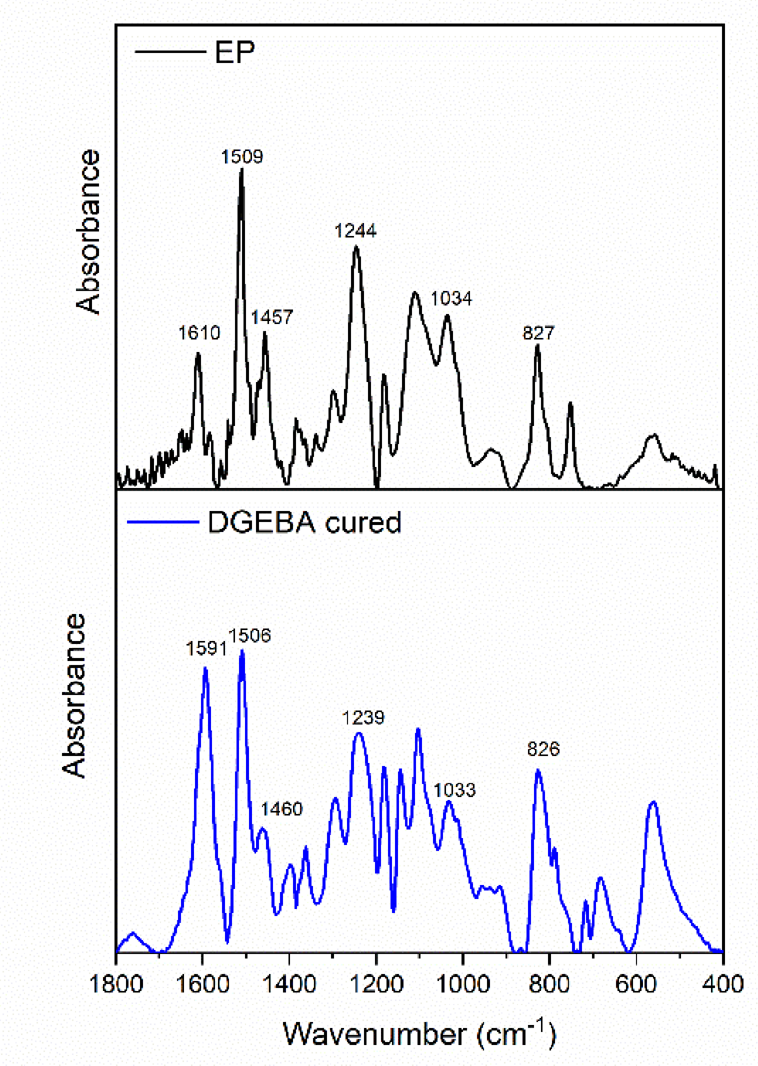


**Figure S4.** Comparison between the FT-IR spectra of the EP sample and an amine-cured DGEBA sample in the wavenumber region of 1800-400 cm$^{-1}$.

Table S7 shows the FT-IR signals assignment.

**Table S7.** FT-IR signals assignment.

| Sample | Stretching vibrations of benzene ring (cm$^{-1}$) | Stretching vibrations of aryl ether unit (cm$^{-1}$) |
|---|---|---|
| EP | 1610-1509-1457-827 | 1244-1034 |
| DGEBA cured | 1591-1506-1460-826 | 1239-1033 |

*Section 4*

*Nuclear Magnetic Resonance (NMR) Spectroscopy*

NMR spectra were recorded on a Bruker AVANCE 400 spectrometer (400 MHz for $^{1}H$, 400 MHz for $^{13}C$, and 400 MHz for DEPT 135). The samples were solubilized in deuterated methanol ($CD_3OD$). The $^{1}H$ and $^{13}C$ NMR chemical shifts are referenced to tetramethylsilane ($Me_4Si$) (δ = 0 ppm), using the residual impurities of the deuterated solvents as an internal standard.

The structure of the degradation products was also explored through $^{1}H$ NMR and $^{13}C$ NMR (see Figures S5 – S6). It was found that signals from the benzene ring and the glycidyl ether unit are present in the degradation products, confirming that depolymerization preserves the main structure of the resin. The $^{1}H$-NMR spectrum (see Figure S5) displays peaks between 7.3 and 6.2 ppm (7.29, 7.13, 6.96, 6.25, and 6.19 ppm), corresponding to the protons of the benzene ring. This indicates that the structure of the benzene ring remains intact in the recycled degradation products. Furthermore, the peaks at 4.05 and 3.51 ppm, corresponding to $CH_2$ groups, the peak at 3.65 ppm, attributed to CH groups, and the peak at 1.13 ppm, assigned to methyl groups ($CH_3$), indicate the presence of the aryl alkyl ether unit (C–O–C) in the degradation products. The evidence that the selective C–N bond cleavage involves the nitrogen atom and the $C_1$ carbon atom, according to the mechanism proposed in Figure 9 of the manuscript, could be the presence of the peaks at 1.99 ppm, assigned to protons of methyl groups bonded to carbonyl group, together with the signal at 1.63 ppm, probably belonging to the proton of –NH secondary amine group.

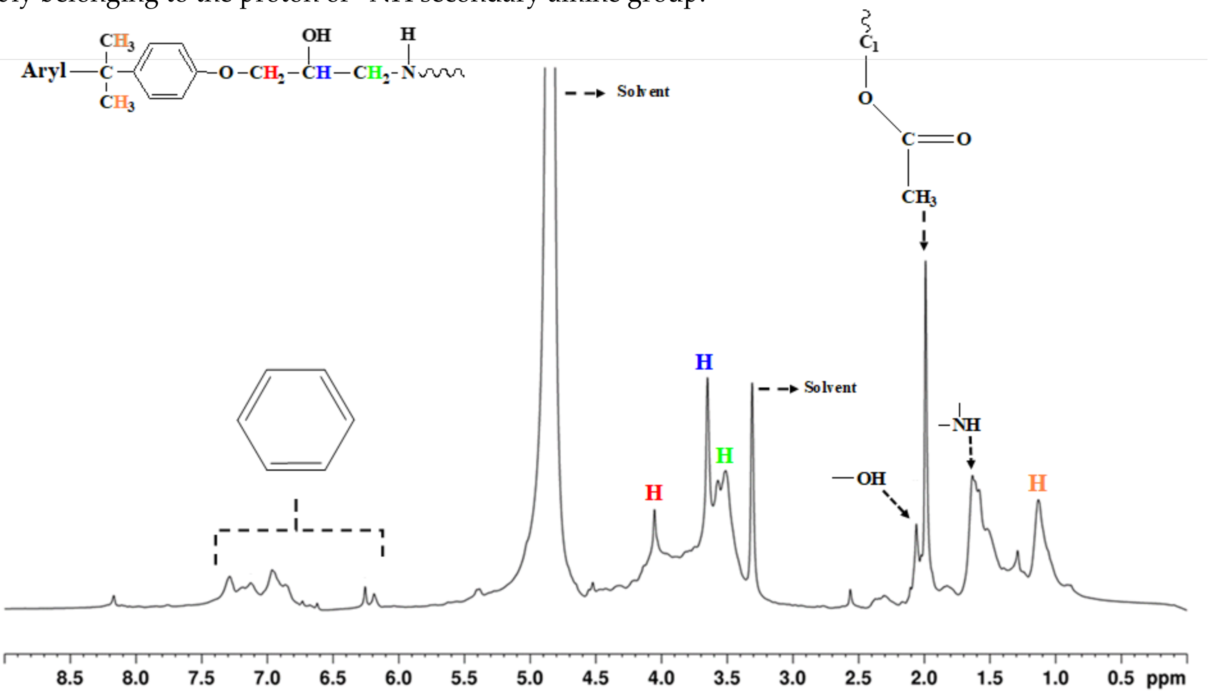


**Figure S5.** $^{1}H$-NMR spectrum of the degradation product DEP.

The assignment of the signals observed in the $^{13}C$ NMR spectrum (see Figure S6) was performed based on the results obtained using the DEPT 135 spectroscopic technique (see Figure S7).

DEPT (Distortionless Enhancement by Polarization Transfer) is an NMR spectroscopy technique that enables the differentiation between methyl, methylene, methine groups, and quaternary carbon atoms. Quaternary carbon atoms do not give signals in DEPT spectra. Three types of DEPT experiments differ in the sequence of pulses used, and each experiment provides different information. The most useful of all is DEPT 135, especially when the $^{13}C$-NMR spectrum is rich in signals. Through DEPT 135, the signals of CH and $CH_3$ groups are positive, that is, they are «above» the baseline, while the signals of $CH_2$ groups are negative, that is, «below» the baseline: the CH and $CH_3$ signals are in «opposition of phase» with respect to $CH_2$. Based on this comparison, in the $^{13}C$-NMR spectrum, the peaks at 135.5, 129.6, 127.5, and 114.6 ppm were assigned to the carbon signals of the benzene ring, the peaks between 80 and 60 ppm were attributed to CH and $CH_2$

carbons of glycidyl ether, the signal at 40.0 ppm was assigned to quaternary carbon of the epoxy precursor and the peak around 20 ppm was ascribed to the methyl groups bonded to the quaternary carbon.

These results highlight that the carbonaceous skeleton of oligomers remains intact in the recycled depolymerization products. Furthermore, the presence of the peak at 174.1 ppm, assigned to the quaternary carbon of the ester carbonyl group, the signal around 30 ppm, attributed to the methyl carbon bonded to the carbonyl group, and a peak around 26 ppm, most probably belonging to a carbon of the $CH_2$ group bonded to a nitrogen atom, could further indicate the selective cleavage of the $C^1$-N bond.

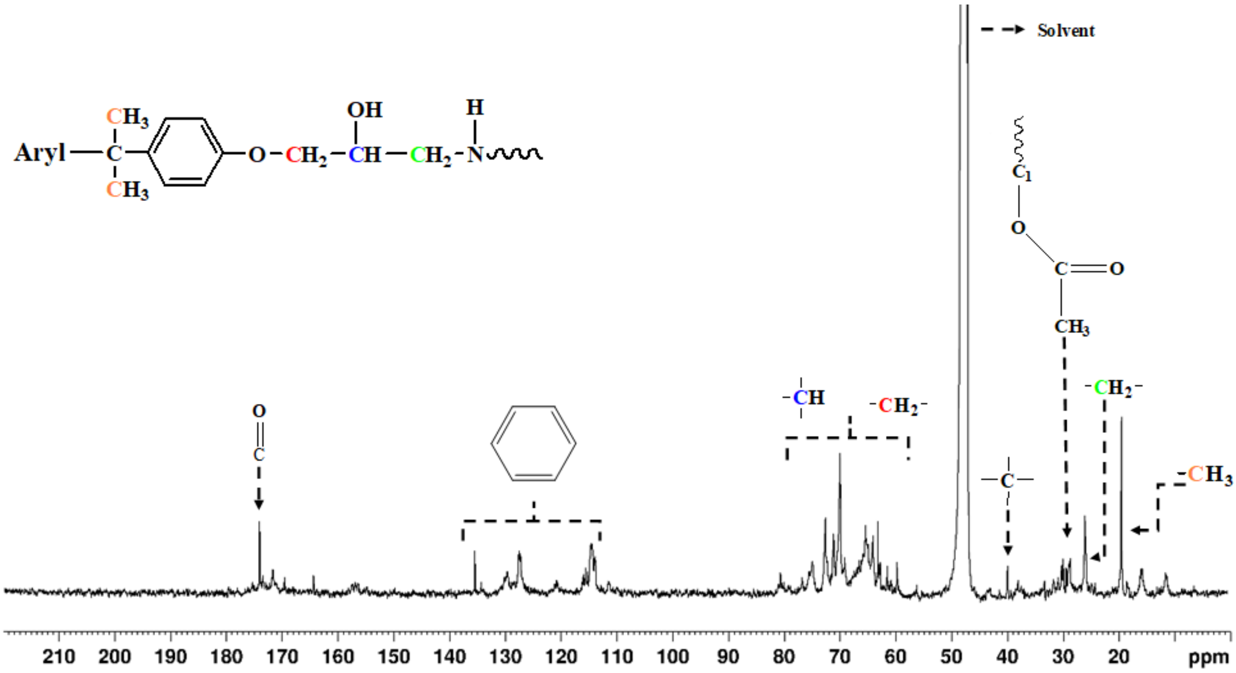


**Figure S6.** $^{13}$C-NMR spectrum of the degradation products DEP.

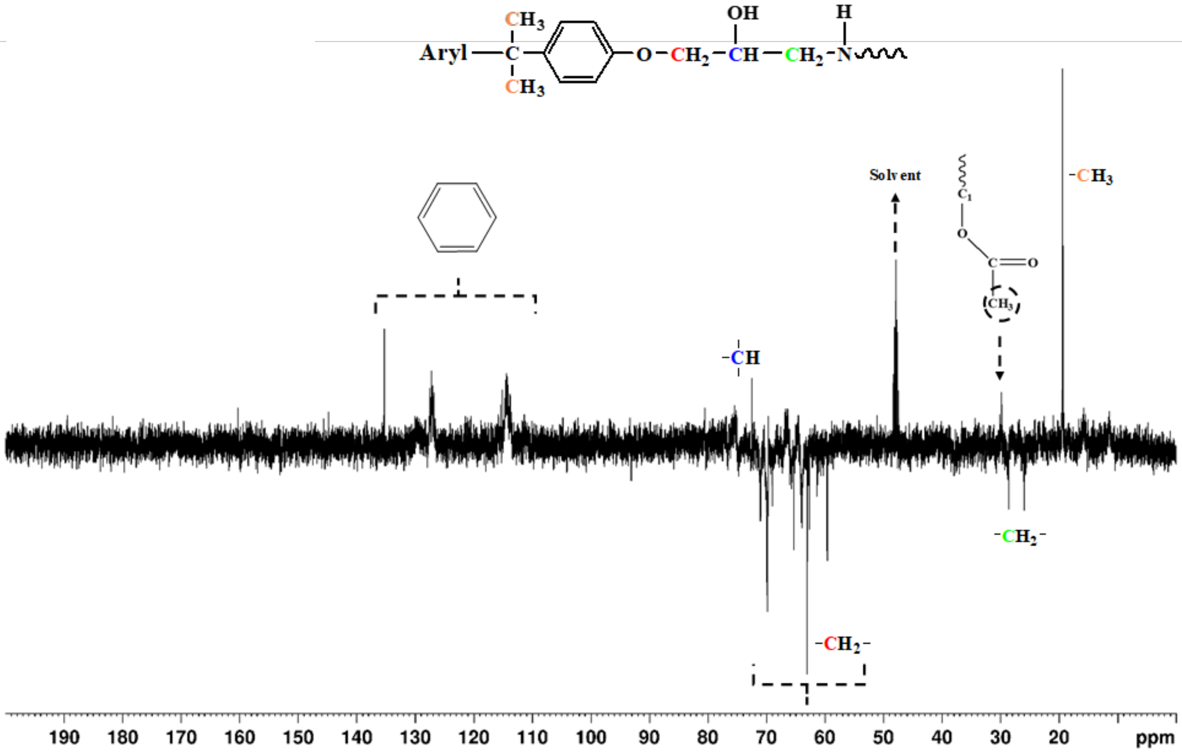


**Figure S7.** DEPT 135 spectrum of the degradation products DEP.
